# Comparison of Working Conditions and Prevalence of Musculoskeletal Symptoms among Dairy Farmers in Southern Sweden over a 25-Year Period

**DOI:** 10.3389/fpubh.2016.00098

**Published:** 2016-05-19

**Authors:** Stefan Pinzke

**Affiliations:** ^1^Department of Work Science, Business Economics and Environmental Psychology, Swedish University of Agricultural Sciences, Alnarp, Sweden

**Keywords:** musculoskeletal symptoms, survey, physical exposure, ergonomics, agriculture, dairy farming

## Abstract

Working conditions and the prevalence of perceived musculoskeletal symptoms (MSSs) among dairy farmers in 2013 were monitored by repeating a mail survey of dairy workers in Scania, southern Sweden, using the same method for collecting data on MSSs and working conditions employed in previous surveys conducted in 1988 and 2002. All dairy enterprises in Scania (total 419) were sent two copies of a questionnaire. One or more responses were received from 232 enterprises (55.4%), of which those from 247 dairy farmers (75% men and 25% women) in 199 enterprises are included in this study. The farmers had increased their weekly working hours in 2013 compared with 2002 (males $\bar{x}=\;43.9,\;40.7$; females $\bar{x}=\;37.9,\;33.9$). Each male milked on average 30 cows in 1988, 44 cows in 2002, and 86 cows in 2013. The corresponding numbers milked by female farmers were 29, 60, and 102, respectively. In 1988, almost all farmers used tethered systems, while in 2013, 54.4% of male and 66.1% of female farmers instead worked with loose-housing systems. Of the farmers who used loose-housing systems, 50.7% had a robotic milking system. In 2013, 79.0% of male and 88.5% of female farmers reported MSSs on some occasion, especially in the lower back, shoulders, and knees for men, and in the shoulders, lower back, and wrists/hands for women. However, there was no statistical change compared with the frequency of MSSs in 2002. In 2013, there was a tendency for younger dairy farmers (≤35 years) to report MSSs, especially in the shoulders, elbows, lower back, and feet, more frequently than younger farmers in 2002. The males who worked with robot milking systems in 2013 indicated less discomfort in the shoulders than men who worked with other systems. The corresponding females indicated fewer problems in the lower back in 2013. Various aspects of milking system design and technology have been improved to reduce the workload and prevent MSSs in dairy farmers. Nevertheless, more improvements are needed to make the milking process more attractive and reduce health problems, especially in younger farmers currently working with milking and in new recruits.

## Introduction

### Dairy Farming and Musculoskeletal Symptoms

Dairy farming in the developed countries worldwide has undergone intensive rationalization over recent decades, leading to fewer operations but larger herd size (1–4). Along with this rationalization, there has been a transition from manual milking in tethered (stanchion) systems to machine milking in loose-housing systems. In tethered systems, which are often used in small-scale dairy farms with smaller herd size (5, 6), the cows are tethered in separate stalls while they are milked. The dairy farmer brings the milking equipment to the cows and stands in between them, kneeling or squatting to perform the work (5, 7). In small-scale dairy farms, it is often the farmer himself who also has to perform other strenuous tasks in addition to milking, such as manual scraping of manure, handling of feed, strewing of litter, and cleaning (8). In loose-housing systems that are more popular among larger dairy farms (7, 9), the milking takes place in a dedicated facility where the milking equipment is stationary. The farm worker performs the milking tasks standing in a more upright posture, either in a milking pit below the cows or at a rotary where the cows pass by on an elevated platform (5, 7, 9). In large-scale dairy farms, the workers are often assigned specific farm operations, such as milking, doing the same highly repetitive and specialized tasks for an entire work shift (8–11).

Automatic milking systems where the milking is performed by robots in milking stations, without depending on human labor, have been used for 20 years in Europe, but have only recently become more popular in North America, in smaller herds with one station and in larger herds with several robotic stations (7, 12, 13).

It is well documented that the milking work in tethered systems is physically demanding, associated with lifting heavy objects, moving and carrying equipment, and awkward working postures, all which are risk factors for development of musculoskeletal symptoms (MSSs), especially in the shoulders, lower back, and knees (5, 10, 14, 15). The repetitive and monotonous milking work in loose-housing systems is considered to pose risk factors for developing MSSs in the upper extremities, especially in the shoulders and wrists/hands (8–10, 15–20).

### Swedish Conditions

In 1990, there were 25,921 farm businesses with dairy cows in Sweden. However, by 2000, this number had fallen to 12,676, in 2010 to 5619, and in 2013 down to 4668 businesses. The average herd size increased over the period, from 22 cows in 1990 to 34 cows in 2000, 62 cows in 2010, and 74 cows in 2013 (21–24).

Most large dairy herds (both in numbers and as a percentage) are located in the province of Scania in southern Sweden. The number of herds in Scania with more than 75 cows increased from 76 in 1990 to 130 in 2000 and to 179 in both 2010 and 2013, while the total number of dairy farms in Scania decreased in those years from 2718 to 1198, 510, and 419, respectively (21–24).

Earlier studies in 1988 and 2002 on dairy farmers in Scania showed that the rationalization described above, along with mechanization and automation of the work, had resulted in a change in pattern concerning working conditions and health for individual farmers (14, 15). In 2002, 83% of male and 90% of female dairy farmers surveyed in Sweden reported some form of perceived MSSs during the previous 12 months. This was an increase compared with the survey in 1988, especially as regards problems in the neck, shoulders, and wrists/hands. By 2002, milkers had increased, on average, their working hours per week, the number of cows milked, and the use of more milking units (15).

In 1988, almost all dairy farmers were working in traditional tethered systems, whereas in 2002, about 25% were working in loose-housing systems (15).

Most dairy farmers in both the 1988 and 2002 survey, irrespective of age or sex, thought that silage handling and milking were their most strenuous tasks. However, the milkers derived their greatest pleasure from the actual milking task, as well from their work with caring for the animals (15).

Overall, the earlier studies (14, 15) showed that individual factors, such as sex, age, and weight, as well as those factors related to work organization and the physical workplace, such as number of hours worked per week, number of cows milked, the milking system used, and the age of the farm building, had significant impacts on the prevalence of MSSs.

The primary aim of the present study was to monitor the current prevalence of MSSs, individual conditions, and the work situation among Scanian dairy farmers by repeating the previous surveys from 1988 and 2002. The objective was to clarify trends on the prevalence of MSSs and the effects on farmers of an additional 10 years of exposure to their work environment, especially to the risk factors found in the previous surveys. The secondary aim was to describe some good practices and technical aids and solutions that can be adopted in different milking systems managed by dairy farmers in order to reduce the workload and prevent MSSs.

## Materials and Methods

The same questionnaire as was used in 1988 and 2003 was employed in the present survey. It comprised questions on perceived MSSs based on the standardized Nordic Musculoskeletal Questionnaires (25), as well as questions about personal characteristics and working conditions. These included items such as the number of cows milked per day, the milking system used, technical aids, occurrence of injuries and health problems beside MSSs, degree of mechanization of the work, which work task the respondents considered to be the most strenuous and which gave the most job satisfaction (26, 27). The questions used regarding MSSs were whether the respondents at some time had (yes/no) perceived ache, pain, or discomfort in the neck, shoulder, elbow, wrist/hand, upper back, lower back, hip, knee, and/or feet during the previous 12 months.

The names and addresses of all dairy farm businesses in Scania (in total 419) listed in the national Farm Register (LBR, 2013) were obtained from the Swedish Board of Agriculture. Each business received two questionnaires by mail in April 2014, enabling two people involved daily, or more regularly, in the milking work (i.e., milkers), e.g., husband and wife, owner and employee, or two employees, to respond. Two reminders were sent out in May 2014 to obtain an acceptable response rate. The first reminder consisted of only a reminder card with a request to complete the questionnaire, whereas with the second reminder, two new questionnaires were sent to those farmers who did not answer the first mailing. In this second mailing, there was also an opportunity to indicate the reason for not participating in the survey.

Of the 418 dairy businesses to which the survey was sent (one business did not receive a mailing because it had an address abroad), 232 businesses responded (55.4%) and 33 did not return a completed questionnaire. Of the latter, 14 had ceased production, were not milking, had no cows, or were deceased; 6 cited lack of time; 5 cited other reasons; and 8 did not state any reason why they did not participate in the study. This means that 247 milker responses from 199 farm businesses were treated in the present study and were compared with the data collected in 1988 and 2002 (Table 1).

**Table 1:** **Number of farms and total number of farmers, divided into males and females, included in the surveys in 2013, 2002, and 1988, and response rates to the questionnaire**.

Year	No. of farms	No. of farmers	Male	Female	Response rate (%)
2013	199	247	186	61	55
2002	504	686	494	188	67
1988	1058	1465	1077	388	81

### Data Analysis

Descriptive statistics regarding demographics, working hours, employment, milking systems, herd size, age of farm buildings, and perceived MSSs, represented by number (*n*), frequency (%), mean, SD, range, and statistical tendency and significance, are presented by gender and survey year in Tables 2–4; by gender and MSSs/no MSSs in 2013 in Tables 5 and 6; by gender and milking robot/other systems in 2013 in Table 7; and by gender, age, and survey year in Table 8.

**Table 2:** **Description and comparison of dairy farmers and their work situation in 1988, 2002, and 2013**.

		2013				2002				1988			
		*n*	Mean^e^	SD	Range	*n*	Mean^e/f^	SD	Range	*n*	Mean^e/g^	SD	Range
Age (year)	Males	184	53.5^d^	10.98	21–83	493	49.4^b/d^	11.00	20–79	1077	47.7^c/c^	11.89	15–81
	Females	61	46.3	13.60	19–71	188	47.3	10.60	20–68	388	45.8	10.89	19–75
No. of years as a dairy farmer	Males	185	32.6^d^	12.24	3–70	494	26.6^d/d^	12.21	1–55	1074	26.1^d^	14.16	1–65
	Females	60	21.8	13.36	1–50	186	20.6	10.83	2–57	386	21.3	13.42	1–50
Hours worked per week	Males	182	43.9^b^	16.89	7–119	490	40.7^d/b^	14.58	2–112	1066	36.3^d/d^	12.39	4–85
	Females	60	37.9	15.75	12–100	187	33.9^/a^	13.10	4–70	379	27.7^/d^	10.86	3–88
Body weight (kg)	Males	184	84.2^d^	11.62	58–116	492	82.0^d/b^	10.70	58–135	1067	79.4^d/d^	9.91	42–122
	Females	60	70.2	12.11	50–100	178	69.5	10.75	45–100	377	65.6^/d^	8.77	50–100
Body height (cm)	Males	185	180.9^d^	7.10	157–200	490	179.6^d/b^	6.79	152–200	1069	177.7^d/d^	6.46	150–205
	Females	60	167.3	6.15	150–181	183	166.9	5.72	150–185	382	165.4^/c^	5.81	150–182
Body mass index (kg/m^2^)	Males	183	25.7	3.16	18.6–36.8	488	25.4	2.95	18.2–41.7	1065	25.1^/a^	2.76	17.0–36.8
	Females	60	25.1	3.95	17.9–35.9	177	25.0	3.77	17.6–39.1	375	24.0^/c^	2.97	17.9–34.6
No. of cows milked	Males	185	85.8	72.72	8–650	492	55.7^/d^	44.16	3–320	1077	30.1^/d^	24.74	2–300
	Females	60	102.3	82.39	8–420	188	59.2^/d^	47.23	12–320	386	29.3^/d^	17.98	1–160

*Descriptive values (*n*, mean, SD, and range), divided by sex*.

*^a^*p* < 0.10*.

*^b^*p* < 0.05*.

*^c^*p* < 0.01*.

*^d^*p* < 0.001*.

*^e^Differences between sexes (independent samples *t*-test)*.

*^f^Differences between dairy farmers in 2013 and 2002 (independent samples *t*-test)*.

*^g^Differences between dairy farmers in 2002 and 1988 (independent samples *t*-test)*.

*Significant increases in values between the 2002 and 2013 surveys are marked in red*.

*The superscript “/” separates the significant levels (a, b, c, d) of the tests with respect to sex (test e) and survey years (tests f, g)*.

**Table 3:** **Description and comparison of the dairy farmers and their work situation in 1988, 2002, and 2013**.

			2013		2002		1988	
			*n*	%^e^	*n*	%^e/f^	*n*	%^e/g^
Employment form	Males	Employed	9	5.0^d^	29	6.1^d^	45	4.2
		Self-employed	171	95.0	446	93.9	1032	95.8
	Females	Employed	12	19.7	28	15.5	10	2.6^/d^
		Self-employed	49	80.3	153	84.5	378	97.5
Handedness	Males	Right	175	94.1	445	89.9^/a^	994	92.3
		Left	6	3.2	38	7.7	68	6.3
		Ambidextrous	5	2.7	12	2.4	15	1.4
	Females	Right	57	93.4	176	92.6	359	92.5
		Left	4	6.6	11	5.8	21	5.4
		Ambidextrous	0	0	3	1.6	8	2.1
Housing system	Males	Tethered	83	45.6	365	74.0^/d^	1032	95.8^b/d^
		Loose-housing	87	47.8	115	23.3	24	2.2
		Both	12	6.6	13	2.6	21	1.9
	Females	Tethered	20	33.9	135	71.4^/d^	381	98.2^/d^
		Loose-housing	35	59.3	46	24.3	3	0.8
		Both	4	6.8	8	4.2	4	1.0
Building date	Males	-1969	15	8.2	31	6.4^/d^	332	31.2^/d^
		1970–1979	23	12.6	117	24.0	491	46.2
		1980–1989	28	15.4	110	22.5	240	22.6
		1990–1999	42	23.1	197	40.4		
		2000–2009	53	29.1	33	6.8		
		2010-	21	11.5				
	Females	-1969	4	6.6	11	6.0^/d^	104	27.1^/d^
		1970–1979	9	14.8	44	23.9	190	49.5
		1980–1989	6	9.8	42	22.8	90	23.4
		1990–1999	10	16.4	72	39.1		
		2000–2009	19	31.1	15	8.2		
		2010-	13	21.3				

*Frequency values (*n* and %), divided by sex*.

*^a^*p* < 0.10*.

*^b^*p* < 0.05*.

*^c^*p* < 0.01*.

*^d^*p* < 0.001*.

*^e^Differences between sexes (Mann–Whitney *U* test)*.

*^f^Differences between dairy farmers in 2013 and 2002 (Mann–Whitney *U* test)*.

*^g^Differences between dairy farmers in 2002 and 1988 (Mann–Whitney *U* test)*.

*Significant increases in values between the 2002 and 2013 surveys are marked in red and significant decreases in green*.

*The superscript “/” separates the significant levels (a, b, c, d) of the tests with respect to sex (test e) and survey years (tests f, g)*.

**Table 4:** **Frequency of perceived symptoms [number (*n*) and %] in the musculoskeletal system at some time during the past 12 months among dairy farmers, divided by sex, in 2013, 2002, and 1988**.

		2013		2002		1988	
		*n*	%^e^	*n*	%^e/f^	*n*	%^e/g^
Neck	Males	50	26.9	139	30.8^b^	229	21.3^c/d^
	Females	20	32.8	72	39.1	112	28.9/^b^
Shoulders	Males	71	38.2^c^	198	43.6^c^	366	34.0^c/d^
	Females	39	62.3	107	58.8	166	42.9^/d^
Elbows	Males	30	15.1	93	20.4^b^	189	17.6^b^
	Females	9	14.8	50	27.8^/b^	87	22.5
Wrists/hands	Males	28	15.1^d^	111	24.3^d/b^	172	16.0^d/d^
	Females	30	49.2	85	46.2	131	33.9^/c^
Upper back	Males	17	9.1^b^	51	11.5	91	8.5^b/a^
	Females	12	19.7	28	15.2	47	12.2
Lower back	Males	99	53.2	247	53.6	594	55.5^b^
	Females	31	50.8	86	46.7	188	48.6
Hips	Males	50	26.9	124	27.6^a^	271	25.3
	Females	14	23.0	63	34.4^/a^	100	25.8^/b^
Knees	Males	64	34.4	174	37.7	429	40.0
	Females	21	34.4	61	33.2	145	37.5
Feet	Males	28	15.1	65	14.3	113	10.5^c/b^
	Females	12	19.7	36	19.6	60	15.5
Any body part	Males	147	79.0^a^	397	83.4^b^	872	81.2
	Females	54	88.5	166	89.7	326	84.2^/a^

*^a^*p* < 0.10*.

*^b^*p* < 0.05*.

*^c^*p* < 0.01*.

*^d^*p* < 0.001*.

*^e^Differences between sexes (Pearson chi-square test)*.

*^f^Differences between dairy farmers in 2013 and 2002 (Pearson chi-square test)*.

*^g^Differences between dairy farmers in 2002 and 1988 (Pearson chi-square test)*.

*Significant decreases in values between the 2002 and 2013 surveys are marked in green*.

*The superscript “/” separates the significant levels (a, b, c, d) of the tests with respect to sex (test e) and survey years (tests f, g)*.

**Table 5:** **Description and comparison of dairy farmers with and without reported musculoskeletal symptoms in 2013**.

		Symptoms 2013				No symptoms 2013			
		
		*n*	Mean^e^	SD	Range	*n*	Mean^e/f^	SD	Range
Age (year)	Males	147	53.5^d^	10.96	21–83	37	53.5	11.17	26–72
	Females	54	45.9	13.57	19–70	7	49.3	14.56	29–71
	Total	201	51.5	12.17	19–83	44	52.8	11.68	26–72
No. of years as a dairy farmer	Males	146	32.6^d^	12.15	4–70	39	32.9^b^	12.73	3–60
	Females	53	21.8	13.56	1–50	7	21.9	12.66	2–40
	Total	199	29.7	13.38	1–70	46	31.2	13.20	2–60
Hours worked per week	Males	143	42.6^a^	16.11	7–119	39	48.7^/b^	18.94	20–105
	Females	53	37.6	15.32	12–100	7	40.0	20.0	20–70
	Total	196	41.2	16.01	7–119	46	47.4^/b^	19.13	20–105
Body weight (kg)	Males	145	84.7^d^	11.53	58–116	39	82.3^c^	11.94	59–110
	Females	53	70.4	12.25	50–100	7	68.7	11.76	55–91
	Total	198	80.9	13.31	50–116	46	80.2	12.77	55–110
Body height (cm)	Males	147	181.0^d^	7.10	157–200	38	180.4^d^	7.14	170–193
	Females	53	167.5	6.30	150–181	7	165.9	5.05	160–172
	Total	200	177.4	9.13	150–200	45	178.2	8.65	160–193
Body mass index (kg/m^2^)	Males	145	25.9	3.10	19.9–36.8	38	25.2	3.38	18.6–35.3
	Females	53	25.1	4.04	17.9–35.9	7	25.0	3.77	20.2–30.8
	Total	198	25.7	3.39	17.9–36.8	45	25.2	3.35	18.6–35.3
No. of cows milked	Males	146	82.6^b^	60.18	8–360	39	97.5	107.75	12–650
	Females	54	104.3	85.35	8–420	6	84.2	49.34	25–150
	Total	200	88.5	68.35	8–420	45	95.7	101.61	12–650

*Descriptive values (*n*, mean, SD, and range), divided by sex*.

*^a^*p* < 0.10*.

*^b^*p* < 0.05*.

*^c^*p* < 0.01*.

*^d^*p* < 0.001*.

*^e^Differences between sexes (independent samples *t*-test)*.

*^f^Differences between dairy farmers with and without musculoskeletal symptoms in 2013 (independent samples *t*-test)*.

*Significant decreases in values between no symptoms and symptoms are marked in green*.

*The superscript “/” separates the significant levels (a, b, c, d) of the tests with respect to sex (test e) and farmers with and without symptoms (test f)*.

**Table 6:** **Description and comparison of dairy farmers with and without musculoskeletal symptoms in 2013**.

			Symptoms 2013		No symptoms 2013	
			*n*	%^e^	*n*	%^e/f^
Employment form	Males	Employed	8	5.6^c^	1	2.7
		Self-employed	135	94.4	36	97.3
	Females	Employed	12	22.2	0	0.0
		Self-employed	42	77.8	7	100.0
	Total	Employed	20	10.2	1	2.3
		Self-employed	177	89.8	43	97.7
Handedness	Males	Right	137	93.2	38	97.4
		Left	5	3.4	1	2.6
		Ambidextrous	5	3.4	0	2.4
	Females	Right	51	94.4	6	85.7
		Left	3	5.6	1	14.3
		Ambidextrous	0	0.0	0	0.0
	Total	Right	188	93.5	44	95.7
		Left	8	4.0	2	4.3
		Ambidextrous	5	2.0	0	0.0
Housing system	Males	Tethered	66	46.2	17	43.6
		Loose-housing	68	47.6	19	48.7
		Both	9	6.3	3	7.7
	Females	Tethered	18	30.0	2	33.3
		Loose-housing	31	62.0	4	66.7
		Both	4	8.0	0	0.0
	Total	Tethered	84	42.9	19	42.2
		Loose-housing	99	50.5	23	51.1
		Both	13	6.6	3	6.7
Building date	Males	-1999	90	62.9^a^	18	46.2^/a^
		2000-	53	37.1	21	53.8
	Females	-1999	26	48.1	3	42.9
		2000-	28	51.9	4	57.1
	Total	-1999	116	58.9	21	45.7
		2000-	81	41.1	25	54.3

*Frequency values (*n* and %), divided by sex*.

*^a^*p* < 0.10*.

*^b^*p* < 0.05*.

*^c^*p* < 0.01*.

*^d^*p* < 0.001*.

*^e^Differences between sexes (Pearson chi-square test)*.

*^f^Differences between dairy farmers with and without musculoskeletal symptoms in 2013 (Pearson chi-square test)*.

*Significant increases in values between no symptoms and symptoms are marked in red and significant decreases in green*.

*The superscript “/” separates the significant levels (a, b, c, d) of the tests with respect to sex (test e) and farmers with and without symptoms (test f)*.

**Table 7:** **Frequency in 2013 of perceived symptoms [number (*n*) and %] in the musculoskeletal system at some time during the past 12 months among dairy farmers, divided by sex, working with a milking robot and other systems**.

		Milking robot		Other system	
		*n*	%^e^	*n*	%^e/f^
Neck	Males	10	19.6	39	29.8
	Females	5	26.3	15	37.5
	Total	15	21.4	54	31.6
Shoulders	Males	14	27.5^a^	54	41.2^c/a^
	Females	10	52.6	27	67.5
	Total	24	34.3	81	47.4^/a^
Elbows	Males	10	19.6	18	13.7
	Females	3	15.8	6	15.0
	Total	13	18.6	24	14.0
Wrists/hands	Males	8	15.7^b^	18	13.7^d^
	Females	9	47.4	21	52.5
	Total	17	24.3	39	22.8
Upper back	Males	4	7.8	13	9.9^b^
	Females	2	10.5	10	25.5
	Total	6	8.6	23	13.5
Lower back	Males	26	51.0	70	53.4
	Females	5	26.3	25	62.5^/b^
	Total	31	44.3	95	55.6
Hips	Males	9	17.6	38	29.0
	Females	4	21.1	10	25.0
	Total	13	18.6	48	28.1
Knees	Males	21	41.2	41	31.3
	Females	6	31.6	15	35.0
	Total	27	38.6	55	32.2
Feet	Males	7	13.7	21	15.3
	Females	3	15.8	9	22.5
	Total	10	14.3	29	17.0
Any body part	Males	39	76.5	104	79.4
	Females	17	89.5	36	90.0
	Total	56	80.0	140	81.9

*^a^*p* < 0.10*.

*^b^*p* < 0.05*.

*^c^*p* < 0.01*.

*^d^*p* < 0.001*.

*^e^Differences between sexes (Pearson chi-square test)*.

*^f^Differences between dairy farmers working with and without milking robot in 2013 (Pearson chi-square test)*.

*Significant decreases in values between symptoms in other symptoms and symptoms in robot systems are marked in green*.

*The superscript “/” separates the significant levels (a, b, c, d) of the tests with respect to sex (test e) and farmers with and without milking robot systems (test f)*.

**Table 8:** **Frequency in 2013, 2002, and 1988 of perceived symptoms [numbers (*n*) and %] in the musculoskeletal system at some time during the past 12 months among dairy farmers, divided by sex and age**.

		2013						2002						1988					
		≤35 years		36–54 years		≥55 years		≤35 years		36–54 years		≥55 years		≤35 years		36–54 years		≥55 years	
		*n*	%	*n*	%	*n*	%	*n*	%^e^	*n*	%^e^	*n*	%^e^	*n*	%^f^	*n*	%^f^	*n*	%^f^
Neck	Males	4	30.8	22	31.9	24	23.5	14	24.1	71	30.1	53	34.2^a^	22	11.4^b^	118	22.6^b^	89	24.8^b^
	Females	7	43.8	9	32.1	4	23.5	9	39.1	44	40.0	18	36.7	20	23.3	69	31.8	23	27.4
	Total	11	37.9	31	32.0	28	23.5	23	28.4	115	33.2	71	34.8^b^	42	15.1^c^	187	25.3^c^	112	25.3^b^
Shoulders	Males	5	38.5	26	37.7	40	39.2	18	30.0	93	39.9	84	53.2^b^	38	19.7^a^	187	35.8	141	39.3^c^
	Females	12	75.0	14	50.0	12	70.6	14	58.3	65	60.7	27	55.1	35	40.7	101	46.5^b^	30	35.7^b^
	Total	17	58.6	40	41.2	52	43.7	32	38.1^a^	158	46.5	111	53.6^a^	73	26.2^b^	288	38.9^b^	171	38.6^d^
Elbows	Males	2	15.4	14	20.3	12	11.8	2	3.4	51	21.3	39	25.2^c^	13	6.8	106	20.3	70	19.6
	Females	2	12.5	5	17.9	2	11.8	0	0.0	39	36.4^a^	11	22.9	10	11.6	60	27.6	17	20.2
	Total	4	13.8	19	19.6	14	11.8	2	2.4^b^	90	26.0	50	24.6^c^	23	8.3^a^	166	22.4	87	19.7
Wrists/hands	Males	2	15.4	14	20.3	12	11.8	13	22.8	55	23.0	43	27.4^c^	31	16.1	78	14.9^c^	63	17.6^b^
	Females	10	62.5	12	42.9	8	47.1	9	37.5	52	47.7	22	44.9	29	33.7	75	34.6^b^	27	32.1
	Total	12	41.4	26	26.8	20	16.8	22	27.2	107	30.7	65	31.6^c^	60	21.6	153	20.7^d^	90	20.4^c^
Upper back	Males	2	15.4	7	10.1	8	7.8	9	16.1	28	12.0	13	8.6	18	9.4	42	8.0^a^	31	8.7
	Females	4	25.0	6	21.4	2	11.8	3	12.5	19	17.4	6	12.2	10	11.6	29	13.4	8	9.6
	Total	6	20.7	13	13.4	10	8.4	12	15.0	47	13.7	19	9.5	28	10.1	71	9.6^b^	39	8.9
Lower back	Males	8	61.5	43	62.3	48	47.1	25	47.1	131	54.8	89	56.0	92	48.4	305	58.3	197	55.0
	Females	10	62.5	15	53.6	6	35.3	10	41.7	55	50.9	20	60.0	37	43.0	112	51.6	39	46.4
	Total	18	62.1	58	59.8	54	45.4	35	41.7^a^	186	53.6	109	52.2	129	46.7	417	56.4	236	53.4
Hips	Males	2	15.4	18	26.1	30	29.4	9	15.3	68	28.6	46	30.7	21	11.0	137	26.2	113	31.6
	Females	1	6.2	8	28.6	5	29.4	3	12.5	38	35.5	21	42.0	12	14.0	63	29.0	25	29.8
	Total	3	10.3	26	26.8	35	29.4	12	14.5	106	30.7	67	33.5	33	11.9	200	27.0	138	31.2
Knees	Males	5	38.5	27	39.1	32	31.4	20	33.9	79	33.3	74	45.7^b^	72	37.3	200	38.3	157	43.9
	Females	5	31.2	8	28.6	8	47.1	6	25.0	36	33.3	18	36.0	22	25.6	83	38.2	40	47.6
	Total	10	34.5	35	36.1	40	33.6	26	31.3	115	33.3	92	43.4^a^	94	33.7	283	38.3	197	44.6
Feet	Males	2	15.4	13	18.8	13	12.7	6	10.2	28	11.7	31	20.4	17	8.8	63	12.0	33	9.2^d^
	Females	4	25.0	6	21.4	2	11.8	1	4.2	25	23.4	9	17.6	4	4.7	38	17.5	18	21.7
	Total	6	20.7	19	19.6	15	12.6	7	8.4^a^	53	15.3	40	19.7	21	7.5	101	13.6	51	11.6^c^
Any body part	Males	11	84.6	56	81.2	80	78.4	50	82.0	200	82.3	144	85.2	140	72.5	441	84.3	291	81.3
	Females	14	87.5	25	89.3	15	88.2	21	87.5	99	90.8	44	88.0	70	81.4	189	87.1	67	79.9
	Total	25	86.2	81	83.5	95	79.8	71	83.5	299	84.9	188	85.8	210	75.3	630	85.1	358	81.0

*^a^*p* < 0.10*.

*^b^*p* < 0.05*.

*^c^*p* < 0.01*.

*^d^*p* < 0.001*.

*^e^Differences between dairy farmers in 2013 and 2002 (Pearson chi-square test)*.

*^f^Differences between dairy farmers in 2002 and 1988 (Pearson chi-square test)*.

*Significant increases in values between the 2002 and 2013 surveys are marked in red and significant decreases in green*.

For statistical analysis of the results, independent samples *t*-tests, Mann–Whitney *U* tests, and chi-square analyses were applied using SPSS version 22 (28). If one cell contained an expected count <5, Fisher’s exact test was used. Otherwise, Pearson’s chi-square was calculated. The probability limits for evaluating statistical tendency (^a^) and significance (^b,c,d^) were ^a^*p* < 0.10, ^b^*p* < 0.05, ^c^*p* < 0.01, and ^d^*p* < 0.001. Significant increases in values between the 2002 and 2013 surveys are marked in red in the tables and significant decreases in green.

### Ethical Considerations

Ethical approval of the Regional Ethical Review Board for studies involving humans was not considered necessary for the survey. The questionnaire was completed anonymously, meaning that no individual or workplace affiliation could be identified. Processing of personal data was performed according to the Personal Data Act (Swedish Code of Statutes, SFS 1998:204), the purpose of which is to protect the individual’s integrity. Overall, the national guidelines based on the World Medical Association Declaration of Helsinki concerning research ethics (29), anonymity, voluntariness, confidentiality, and archiving of data were considered and fulfilled.

## Results

### Demographics, Working Hours, Employment, Milking Systems, Herd Size, and Age of Farm Buildings

Of the total of 247 respondents in 2013, 186 (75.3%) were men and 61 (24.7%) were women.

Compared with the female dairy farmers surveyed, male farmers were on average 7 years older ($\bar{x}=\text{ 53}.\text{5},\text{ 46}.\text{3;}$ *p* = 0.000), had worked 11 years longer as a dairy farmer ($\bar{x}=\text{ 32}.\text{6},\text{ 21}.\text{8}$; *p* = 0.000), and worked 6 h more per week ($\bar{x}=\text{ 43}.\text{9},\text{ 37}.\text{9}$; *p* = 0.016) (Table 2).

Both male and female farmers had increased their working hours, by 3 and 4 h/week, respectively, in 2013 compared with 2002 (males $\bar{x}=\text{ 43}.\text{9},\text{ 4}0.\text{7}$; *p* = 0.016; females $\bar{x}=\text{ 37}.\text{9},\text{ 33}.\text{9}$; *p* = 0.055). The men in 2013 were about 3 cm taller and weighed about 2 kg more than the men in 2002 (Table 2). Each male milked on average 30 cows in 1988, 44 cows in 2002, and 86 cows in 2013. The increase between years was significant (*p* = 0.000 and *p* = 0.000, respectively). The corresponding number of cows milked by female farmers in 1988, 2002, and 2013 was 29, 60, and 102, respectively (difference *p* = 0.000 and *p* = 0.000, respectively) (Table 2).

Women were more frequently farm employees (rather than managers/owners) than their male colleagues in 2013 (19.7 vs. 5.0%; *p* = 0.000) (Table 3).

In 1988, almost all farmers used a tethered system and only 4.1% of male farmers worked with a loose-housing system. This figure increased to 25.9% in 2002 and 54.4% in 2013 (*p* = 0.000 and *p* = 0.000, respectively). The corresponding increase for female farmers was from 1.8% in 1988 to 28.5% in 2002 and 66.1% in 2013 (*p* = 0.000 and *p* = 0.000, respectively) (Table 3).

About half (50.7%) of the farmers who stated that they used a loose-housing system had a robotic milking system.

In 2013, more than 40% of men and 50% of women worked in farm buildings built in 2000 or later (Table 3).

### Musculoskeletal Symptoms

About 79.0% of men and 88.5% of women reported MSSs at some time in 2013, whereas in 2002, 83.4% of men and 89.7% of women indicated MSSs. This change was not significant (*p* = 0.187 and *p* = 0.791 for men and women, respectively). As in 2002, in 2013, men more often reported symptoms in lower back (53.2%), shoulders (38.2%), and knees (34.4%). The women surveyed in 2013 most often reported discomfort in shoulders (62.3%), lower back (50.8), and wrists/hands (49.2%). This pattern was the same as in 2002. No significant change in the frequency of MSSs in 2013 compared with 2002 was observed in the three most frequent body regions for either men or women (Table 4).

The men in 2013 who stated that they had experienced trouble in some body region worked an average of 6 h less per week than the men who did not report any such trouble (Table 5). In addition, they worked more often in older buildings (Table 6).

Both men and women reported symptoms at some time in 2013 equally frequently, about 80 and 90% respectively, regardless of whether they worked in a tethered system or loose-housing system (Table 6). However, the men who worked with a milking robot reported significantly fewer symptoms in the shoulders than the men who worked in other systems. The women who worked with a milking robot reported fewer problems in the lower back (Table 7).

The younger dairy farmers (≤35 years) in 2013 more often reported discomfort in the shoulders (*p* = 0.054), elbows (*p* = 0.020), lower back (*p* = 0.058), and feet (*p* = 0.076) compared with 2002, while the older farmers (55 years and older) reported fewer problems with the neck (*p* = 0.034), shoulders (*p* = 0.084), elbows (*p* = 0.005), wrists/hands (*p* = 0.004), and knees (*p* = 0.081) (Table 8).

### Aids and Facilities

**Figure 1:**
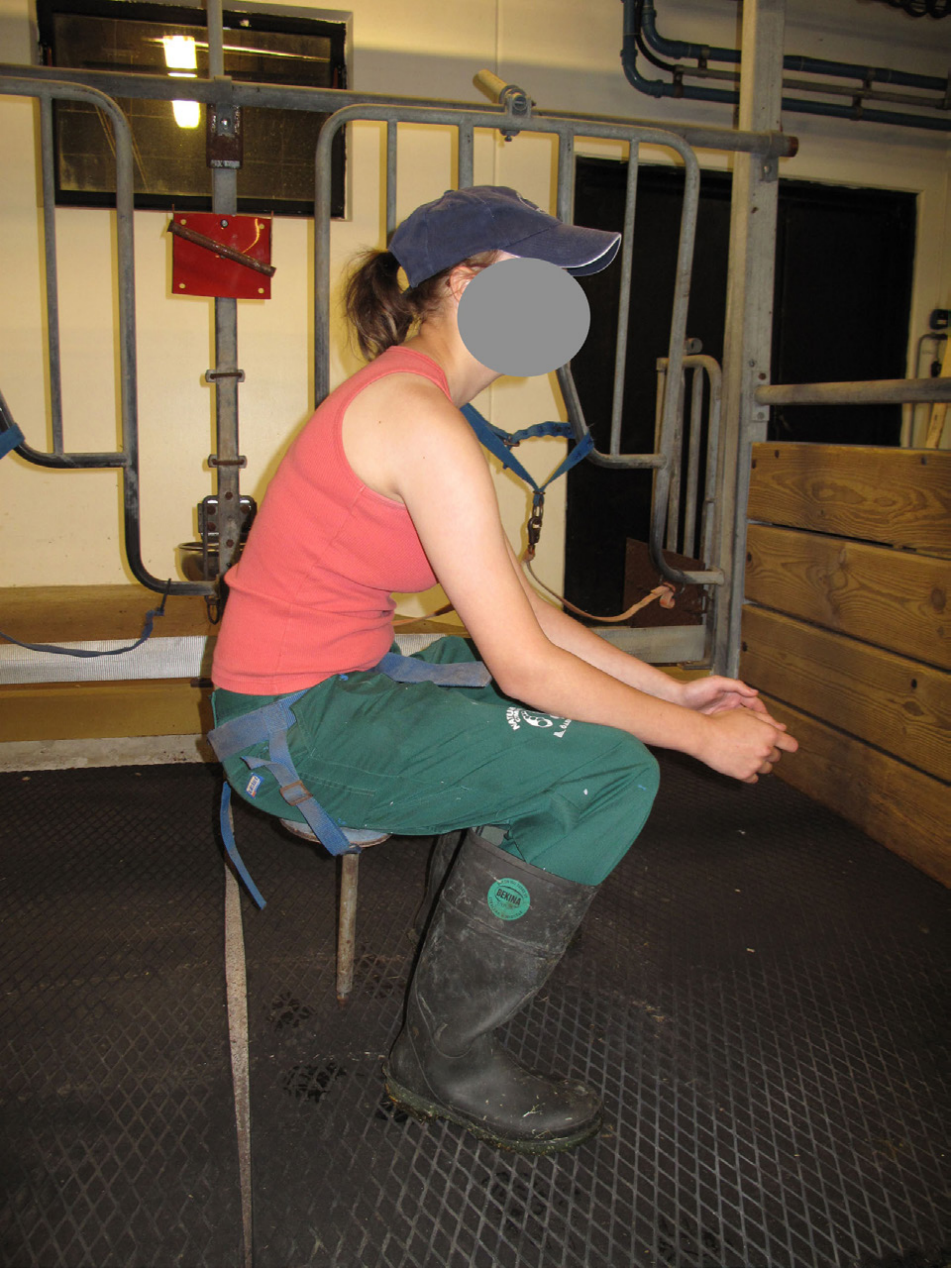
**Milking stool**. ©Christina Lunner Kolstrup.

**Figure 2:**
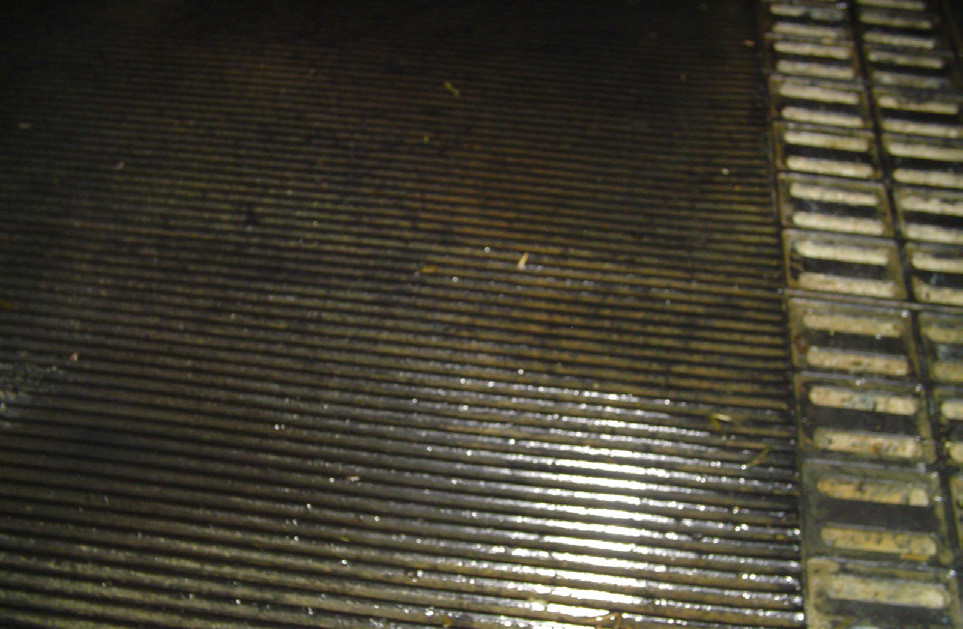
**Rubber matting**. ©Stefan Pinzke.

**Figure 3:**
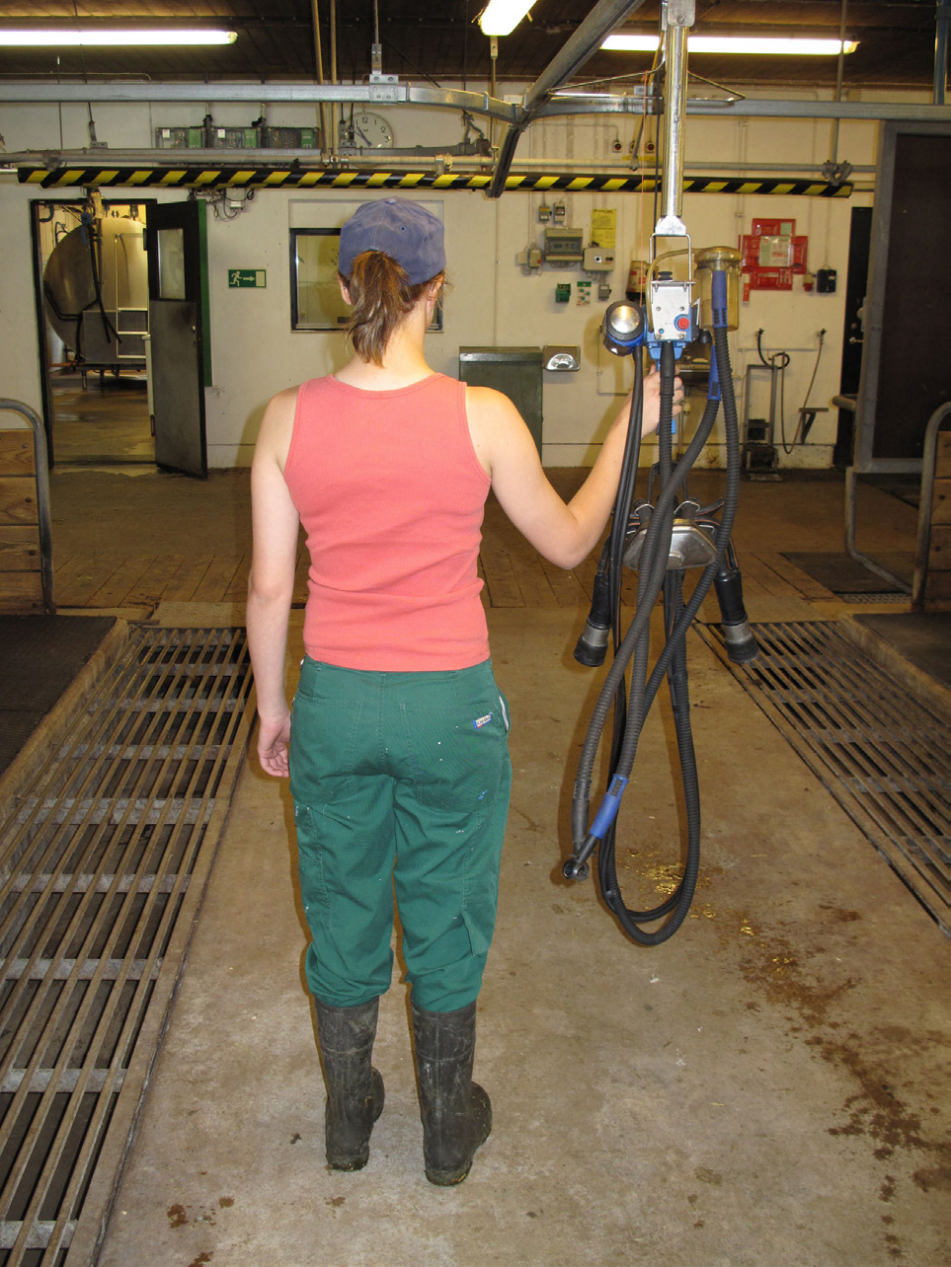
**Milking rail**. ©Christina Lunner Kolstrup.

**Figure 4:**
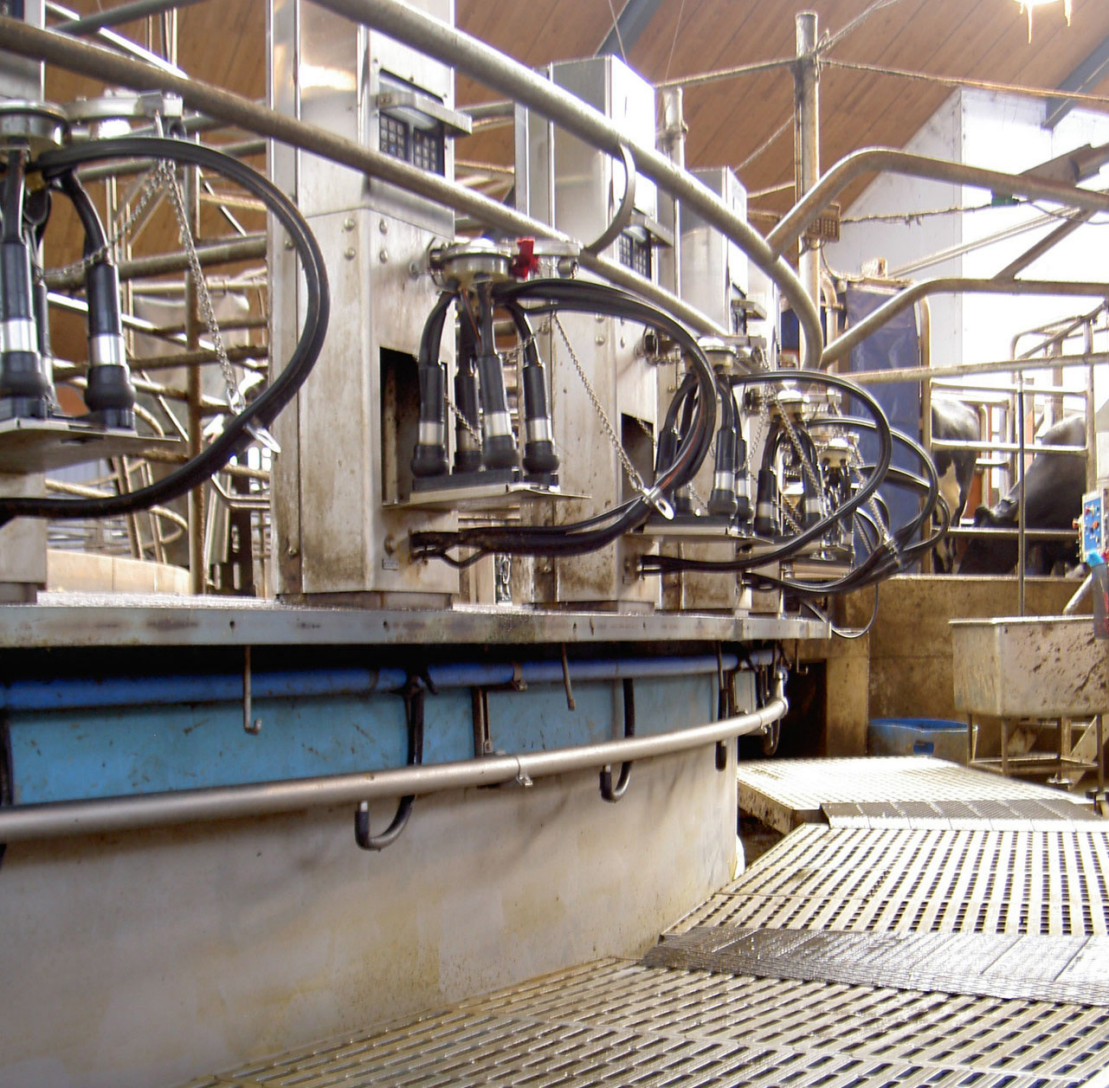
**Automatic cluster removal**. ©Christina Lunner Kolstrup, Stefan Pinzke.

**Figure 5:**
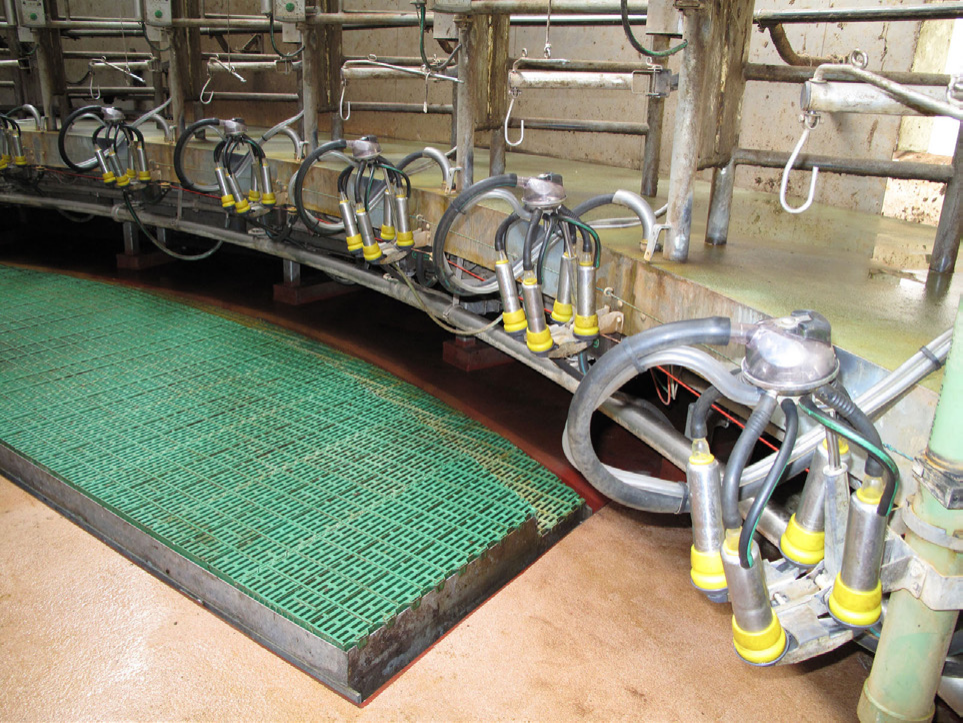
**Adjustable floor**. ©Christina Lunner Kolstrup, Stefan Pinzke.

**Figure 6:**
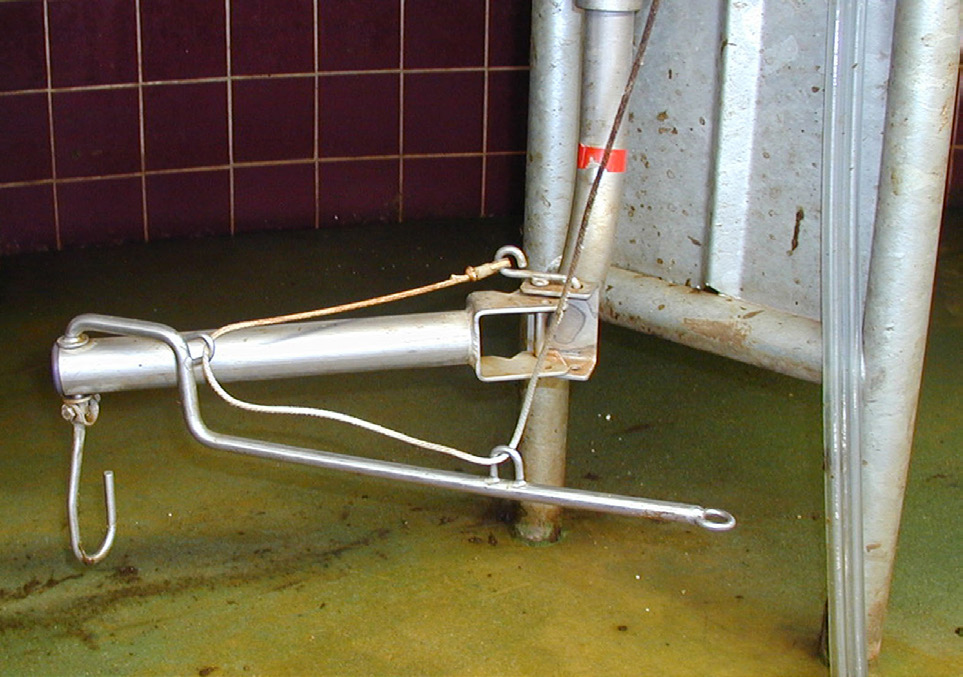
**Support arm**. ©Christina Lunner Kolstrup, Stefan Pinzke.

In the tethered systems, the following facilities were used: milking stool (48.5%) (Figure 1), “kangaroo bag” [a belt to wear containing a bottle holder and large bags for carrying milking towels (28.2%)], rubber mat on the floor (33.0%) (Figure 2), milking rail (36.9%) (Figure 3), and automatic cluster removal (32.0%) (Figure 4). In loose-housing systems, farmers used kangaroo bag (3.3%), rubber mat on the floor (7.4%), automatic cluster removal (39.3%), height-adjustable floor (18.0%) (Figure 5), and support arm (10.7%) (Figure 6).

### Manure and Feed Handling

In loose-housing systems, both manure management and feed handling were more mechanized than in tethered systems. About 86% used a pressure washer in loose housing, compared with 83% in tethered systems.

### Health Problems and Injuries

Approximately 17% of the dairy farmers surveyed indicated that they had health problems other than MSSs arising from their work in tethered systems, compared with 9% in loose-housing systems. Common symptoms were asthma, allergies, and rashes, but also disorders of the respiratory system such as sneezing, coughing, and colds. The dairy farmers also indicated experiencing fatigue and stress.

A total of 32.8% of dairy farmers had suffered some form of injury at work. Among those who worked in tethered systems, 40.8% had experienced an injury, compared with 30.7% in loose-housing systems. Animal-related injuries dominated, such as kicks, trampling, crushing, and butting by the animals. Fall injuries also occurred in both systems.

### Strenuous Duties

Overall, farmers working in tethered systems reported that feed/silage management and milking itself were the most strenuous tasks, while farmers working in loose-housing systems reported cleaning and feeding/handling silage as the most exhausting.

### Job Satisfaction

For farmers using the tethered system, working with the animals and the milking itself gave the most job satisfaction, while for those working in loose-housing systems, working with the animals and calves gave the most job satisfaction.

## Discussion

The results of this most recent survey show that milking dairy cows is still associated with a high incidence of MSSs, as found previously among dairy farmers with smaller herd size operations (8, 16, 30) and operations with larger herd size (9).

No statistically significant reduction in the total number of complaints in 2013 was observed compared with 2002, despite the technological developments that have taken place over the last 20 years. A concerning finding was that young dairy farmers (≤35 years) more frequently reported symptoms than the corresponding young dairy farmers in 2002. However, the dairy farmers who were 55 and older reported fewer complaints than in 2002. This may be because older farmers with health complaints had stopped milking due to their problems in the interim and that only the healthy elderly remained in the profession (the so-called healthy worker effect). This effect was also observed in the 2002 study, where more than 20% of those who had stopped milking cited occupational health reasons for this (15). The effect has also been reported in other studies on musculoskeletal disorders among farmers (31) and among pig keepers with lung problems (32).

One advantage of the present study and of the previous two surveys was the availability of a relatively large body of material collected using the same validated and standardized questionnaire for assessment of MSSs, which made it possible to study trends in the prevalence of MSSs among Scanian dairy farmers. However, it was not possible to grade the severity or the type of MSSs, since the relevant questions in the questionnaire only asked if the respondents had at some time experienced MSSs, and did not enquire about the severity or the type of symptoms. For this, more in-depth studies are needed. Moreover, it was not possible to establish causality between MSSs and the risk factors studied, since the present study and the previous surveys were designed as cross-sectional studies where variables were measured at the same time. Therefore, we could not establish whether the MSSs or exposure to the risk factors came first.

In addition to MSSs, dairy farmers suffer work-related injuries. In 2013, approximately one-third of the dairy farmers in Scania reported that they had been injured during work at some time. A previous study of injuries in agriculture showed that on 15% of Swedish dairy farms, at least one accident occurred in 2004 (33). Preliminary results from an ongoing study on injuries in agriculture in 2013 (Pinzke and Lundqvist, manuscript) show no reduction in the number of injuries compared with 2004 when the number of hours worked is taken into account.

Several studies have shown that compared with milking in parlor systems, milking in tethered stall systems involves more loading work postures and more handling of manual materials, which are risk factors for MSSs in the shoulders and lower part of the body. On the other hand, milking in loose-housing systems involves repetitive and monotonous work, which is a risk factor for developing MSSs, especially in the upper extremities (5, 8–10, 14–20). As this study shows, milkers still reported an equally high frequency of MSSs as in the past, regardless of whether they worked in tethered or parlor systems. However, those working with milking robot systems in 2013 reported fewer MSSs overall, especially in shoulders (men) and lower back (women), compared with those working with other systems. An explanation for this is of course that the robot, instead of the milker, performs most of the heavy, repetitive, and one-sided milking tasks. A reduction in the risk of musculoskeletal problems with robotic milking compared with conventional milking has also been reported in other studies (34). Just over 28% of the Scanian dairy farmers surveyed in 2013 responded that they worked with robotic milking systems. This corresponds fairly well with the incidence of robotic milking (32%) throughout the country (35).

Many developments have been made in technical aids and the design of milking systems in order to reduce workloads and prevent musculoskeletal disorders when milking cows (10, 36). In an EU project where SLU was one partner (37), several good practices were observed on farm visits across Belgium, Poland, Sweden, and UK, e.g., installation of milking rails in tethered houses to facilitate transport of milking equipment and adjusting the floor to the height of the farmer in loose-housing systems. Use of perforated rubber matting on existing floors in parlors is another example of good practice that aims to reduce the physical load on the lower limbs and reduce fatigue. Other solutions are designed for specific tasks during milking in parlors; e.g., when cleaning udders, central placement of a basket for drying papers or cloths on a cart reduces both walking distance and exposure to awkward back postures for the milking staff. Installation of a support arm can reduce the workload when attaching the milking cluster to the cow. The use of lightweight clusters and tubes also reduces the load. Instead of using a dip cup for teat dipping, the farmer can spray the cow’s teats with disinfectant, thus reducing the reach distance during work. Despite these solutions in place on existing farms, not enough research has been done on specific ergonomic interventions in milking parlors.

Some studies have attempted to find the optimum working height for dairy farmers during milking. Jakob et al. (38) found that the optimum working height when attaching teat cups to the udder is having the cow’s teats at shoulder level, while Stål and Pinzke (39) found that the ideal working posture is when the farmer’s elbow height is about 30 cm above the floor where the cow is standing.

The technical aids described above, such as an adjustable floor, support arm, and lightweight clusters, can improve the loading conditions for the farmer if they are applied correctly. However, because of the wide variation in the body composition of cows and differences in the body height of dairy farmers, there is still no technical solution to ensure an optimum working position for all workers at all times.

This study showed that milkers in 2013 were still reporting as many MSSs as 10 years earlier, despite the technical solutions that have been introduced in different milking systems to reduce risk factors for developing MSSs, such as awkward working postures and physical workload. At the same time, exposure to other risk factors has increased, e.g., weekly working hours, number of milking cows, and a higher proportion of working in loose-housing systems, where milkers are exposed to monotonous and repetitive work. Thus, there is a need for continued efforts and research to improve the ergonomic conditions on dairy farms in order to make milking work more attractive, with fewer musculoskeletal problems, especially for younger dairy farmers who are currently working with milking, but also to attract new recruits.

## Author Contributions

The author planned the study, carried out the data collection, analyzed the data, and wrote the article.

## Conflict of Interest Statement

The author declares that the research was conducted in the absence of any commercial or financial relationships that could be construed as a potential conflict of interest.
